# Rapid generation of long tandem DNA repeat arrays by homologous recombination in yeast to study their function in mammalian genomes

**DOI:** 10.1186/1480-9222-13-8

**Published:** 2011-10-07

**Authors:** Vladimir N Noskov, Nicholas CO Lee, Vladimir Larionov, Natalay Kouprina

**Affiliations:** 1 Laboratory of Molecular Pharmacology, National Cancer Institute, National Institutes of Health, 9000 Rockville Pike, Bethesda, Maryland 20892, USA

## Abstract

We describe here a method to rapidly convert any desirable DNA fragment, as small as 100 bp, into long tandem DNA arrays up to 140 kb in size that are inserted into a microbe vector. This method includes rolling-circle phi29 amplification (RCA) of the sequence *in vitro *and assembly of the RCA products *in vivo *by homologous recombination in the yeast *Saccharomyces cerevisiae*. The method was successfully used for a functional analysis of centromeric and pericentromeric repeats and construction of new vehicles for gene delivery to mammalian cells. The method may have general application in elucidating the role of tandem repeats in chromosome organization and dynamics. Each cycle of the protocol takes ~ two weeks to complete.

## Introduction

Tandem repeats, also referred to as satellite DNA, represent a major class of repetitive DNA, whose function in the shaping of the human genome is only beginning to be explored. These repetitive sequences can be located in exons, introns, or intergenic regions, and their polymorphisms provide a unique source of genomic variability. Recent evidence also suggests that the repeat variants can influence the expression of entire loci and disease susceptibility [1,2]. Satellite sequences vary both in their repeat unit size and in their array length. Microsatellites are the smallest, with a repeat size of as little as few base pairs. The expansions of microsatellites associated with diseases have been referred to as dynamic mutations. Another class of repeat sequences is the classic satellites. They are much more abundant in the mammalian genome and consist of larger size repeat units ranging from 20-30 base pairs to a few kilobases. The array size of classic satellites may exceed several megabases.

The most commonly known regions enriched by classic satellites are centromeric and pericentromeric regions. In mammals and other multicellular eukaryotes, these regions are characterized by very large arrays of different tandem repeated DNA sequences. Although centromeric DNA repeat sequences are thought to be structurally and/or functionally important for forming a functional kinetochore, they are poorly conserved between species. For example, mouse chromosomes have two types of DNA repeat sequences, the major satellite repeat (~6 Mb array/234 bp per repeat unit) and the minor satellite repeat (~600 kb array/120 bp per repeat unit) [3,4]. The major mouse satellite is found in the pericentromeric region, and the minor mouse satellite is found in the centric constriction of the centromere [3,5]. The centromeres of human chromosomes are characterized by the presence of megabase-size alpha-satellite DNA arrays (also known as alphoid DNA), which are composed of a tandem array of a 171 bp repeat unit with no homology to mouse minor and major satellites. Moreover, alpha-satellite DNA arrays are diverged between different chromosomes. Non-alphoid DNA repeats have also been identified adjacent to alpha-satellite DNA in the pericentromeric regions of human chromosomes, for example, satellites I, II, and III [6-8], beta-satellite DNA [9], sn5 satellite DNA [3,10], and gamma-satellite DNA [11-14]. Alpha-satellite DNA is the only centromeric DNA sequence identified to date with a proposed role in kinetochore formation and maintenance. Long arrays of alpha-satellite DNA can form kinetochores *de novo *during transfection into human cells [15-21]. However, this process is poorly studied, and untill now the precise function of satellite DNA in centromeres remains mostly undetermined.

Because there is no approach to delete or mutate satellite DNAs in the genomic context, their function can be clarified, for example, by their insertion into ectopic chromosomal site. However, such an approach has limitations because chromosomal regions enriched by satellite DNA are underrepresented in the existing BAC and YAC libraries. Moreover, the monomers of centromeric and pericentromeric arrays in BAC/YAC clones are highly diverged, making their mutational analysis impossible. Several years ago, two groups suggested to study the function of centromeric repeats by the construction of synthetic alphoid arrays using repetitive directional ligation on the basis of a native higher-order 2-3 kb repeat fragment [22,23]. Although this approach allowed the construction of several large synthetic arrays, it has significant limitations. First of all, it is a slow, laborious strategy not easily scaled up for rapid generation of tandem repeats with engineered changes. Secondly, the method uses restriction sites that may not be available in an amplified repeat unit. In addition, artificially introduced restriction sites remain in multiple copies in the final constructs.

To overcome all these problems, we developed another strategy to rapidly assemble long arrays of classic satellite DNA with a size up to 140 kb from a monomer or oligomer. This technique is comprised of two steps: rolling-circle amplification (RCA) of a short DNA multimer (e.g., a dimer for alphoid DNA) into 2-15 kb DNA branched molecules (that are poorly clonable in bacterial vectors) and subsequent assembly of the amplified molecules into long arrays by transformation-associated recombination (TAR) in yeast [24]. As a result, amplified arrays are propagated as circular YAC/BACs in yeast cells and can be transferred into *E. coli *cells if needed. As any nucleotide can be easily changed in the original satellite unit before its amplification, this new technique is optimal for identifying the nucleotides sequence(s) in the satellite DNA critical for a specific function.

Using the RCA-TAR method, we constructed a set of different alphoid DNA arrays that have been used to elucidate the structural requirements for *de novo *kinetochore formation in human cells [24-27]. In addition, a synthetic alphoid DNA array with an embedded tet-operator sequence was used to construct a new generation of Human Artificial Chromosomes (HACs) with a conditional centromere for gene delivery and gene expression studies [25,28]. Being applied for pericentromeric repeats, this method identified the insulator activity of gamma-satellite DNA that prevents heterochromatin spreading beyond the pericentromeric region [29]. This barrier activity may be exploited in gene expression studies to protect transgenes from epigenetic gene silencing. In addition, amplified repeats are also important for experiments on epigenetic engineering. The principle of this approach is based on the insertion of an amplified sequence carrying multiple tet operator (tetO) or lac operator (lacO) sequences into an ectopic chromosomal site that allows the tethering of chromatin modifiers into the array as tet repressor (tetR) or lac repressor (lacR) fusions. Recently, the synthetic tetO-alphoid/tetR-fusion tethering system has been used to clarify the role of open and condensed chromatin in maintenance of the human kinetichore [27]. This system also allowed the induction of *de novo *kinetochore assembly on both newly introduced synthetic alphoid DNA arrays^25 ^and at the ectopic site [30]. Some examples of mammalian satellite DNAs amplified by RCA-TAR are shown in Table 1. This method may also have application for the construction of protein-polymers derived from short peptide motifs found in some proteins to design novel drug delivery vehicles and for high-yield synthesis of peptide drugs and antigens [[31] and references therein].

**Table 1:** Synthetic arrays generated from different types of repeats

Repeat unit	Size of unit	Size of array	Fold increase
*Human alphoid DNA*^24-28^			
2 mer	0.34 kb	40 kb	x118
2 mer/mutant*	0.34 kb	60 kb	x176
4 mer	0.68 kb	70 kb	x103
5 mer	0.85 kb	140 kb	x165
6 mer	1.02 kb	35 kb	x35
tetO-CENP-B/2 mer**	0.34 kb	50 kb	x147
tetO-CENP-B/2 mer**	0.34 kb	50 kb	x147
*Human gamma-satellite DNA*^29^			
1 mer	0.24 kb	24 kb	x100
*Mouse satellite DNA*^29^			
Major, 3 mer	0.7 kb	55 kb	x79
Minor, 4 mer	0.5 kb	10 kb	x20

*Dimer from a chromosome 17 alphoid 16-mer higher order repeat containing a mutated CENP-B biding motif (CENP-B box).

**Novel artificial alphoid dimers composed of one monomer from a chromosome 17 alphoid 16-mer higher order repeat linked to a wholly synthetic alphoid monomer based on a published consensus sequence. The natural monomers contain either a wild type or mutated CENP-B box. In the synthetic monomer, this was replaced with a 42 bp tetracycline operator (tetO), the binding site for *E. coli *tetracycline repressor (tetR).

Collectively, the RCA-TAR-based strategy may be applied for the analysis of any type of repeats in mammals, whose functions are still unclear. Manipulation of the number of DNA repeats with different mutations can be the basis for experiments to elucidate the critical parameters that lead to heterochromatinization or formation of transcriptionally-active chromatin domains for stable transgene expression.

### Experimental design

#### Design of the TAR cloning vector

This vector should be constructed before RCA-TAR cloning experiments. It contains two targeting sequences (or hooks) that are at least 90% homologous to the repeat sequence, and each hook has a minimal length of 60 bp. The hooks are inserted into the polylinker of the TAR vector in a head-to-tail orientation. Before yeast spheroplasts transformation, the TAR cloning vector is linearized by cleaving between the targeting hooks. A unique endonuclease recognition site producing either blunt or cohesive ends is placed between the hooks to allow linearization of the vector and thus 'activate' the hooks.

#### Yeast cells

The current protocol has been optimized for the strain VL6-48. The "recombinational cloning" part of this protocol has been optimized for processing 1.0 × 10^9 ^spheroplasts cell number from early stationary phase cultures grown in 50 ml of YEPD media.

#### Optimization of spheroplasts preparation

While there are no limitations in the choice of a yeast host strain, the time of the Zymolyase-20T treatment or its concentration for each new batch of zymolyase should be experimentally determined, if using different amounts of cells, different phases of cell culture growth, or different yeast strains, because different strains may exhibit a different sensitivity to the enzyme. Each spheroplasts transformation uses 2-4 μg of RCA product and 0.01-0.04 μg of the linearized vector. Typically, under such conditions, 200-5000 transformants are obtained. As a control for recombination, the RCA products are omitted from the transformation mix, resulting in a decreased yield of transformants, down to 5-20 colonies.

#### Confirmation of the repeat amplification

When the arrays are assembled, a control experiment should be carried out to confirm the correct amplification of the repeats in the BAC DNA arrays. For this purpose, several BACs with the largest arrays should be digested by an endonuclease that cuts the arrays until their original repeat units (i.e., a unique endonuclease site present only once in each monomer).

#### Successive size increase of the array

Should the analysis of a representative number of *E. coli *colonies (~60) fail to reveal an array with the desired size, an additional round of recombinational cloning is required to further increase the size of the array. For this purpose, the BAC vector with the largest insert found during the first round of amplification is digested with an appropriate endonuclease that cleaves at the insert/vector junctions. The vector DNA is eliminated with an additional endonuclease that cuts only the vector part into small fragments. The final digest is precipitated with ethanol/sodium acetate and dissolved in a small volume of water. For the second round of yeast spheroplast transformation, usually 2-4 μg of the released BAC-insert array and 0.01-0.02 μg of the TAR vector are required. The yield of clones with a 2- to 3-fold larger insert size is typically 5-10%.

#### Amplification of the repeat unit without the RCA step

Although the RCA reaction accelerates the assembly of the repeats into long arrays, this step may be omitted. Recombinational assembly may be performed using a synthesized oligonucleotide corresponding to a dimer or a tetramer of a repeat of interest, instead of the RCA products.

## Materials

### Reagents

A highly transformable *Saccharomyces cerevisiae *strain VL6-48 (*MAT alpha, his3-D 200, trp1-D1, ura3-52, lys2, ade2-101, met14, psi+cir*^*0*^) that has *HIS3 *and *TRP1 *deleted is used as a host for recombinational cloning experiments. This strain is available from the American Type Culture Collection (ATCC Number MYA-3666™).

MegaX DH10B™ T1R Electrocomp™ Cells (Invitrogen, cat. no. C6400-03)

Bacto yeast extract (Fisher Scientific Ltd., cat. no. DF0886-17-0)

Bacto peptone (Fisher Scientific Ltd., cat. no. DF0118-17-0)

Bacto tryptone (Fisher Scientific Ltd., cat. no. DF0123-07-5)

Bacto Agar (Fisher Scientific Ltd., cat. no. DF0145-17-0)

D-Glucose (Sigma Chemical Co. Ltd., cat. no. G5250-1 KG)

Yeast Nitrogen Base w/o Amino Acids (BD-Diagnostic Systems, cat. no. 291920)

Adenine hemisulfate (Sigma-Aldrich, cat. no. A-3159)

Uracil (Sigma-Aldrich, cat. no U075)

L-Arginine-HCl (Sigma-Aldrich, cat. no. A4881)

L-Aspartic acid (Sigma-Aldrich, cat. no. A93100)

L-Glutamic acid (Sigma-Aldrich, cat. no. 128430)

L-Histidine-HCl (Sigma-Aldrich, cat. no. 1515668)

L-Isoleucine (Sigma-Aldrich, cat. no. 151718)

L-Leucine (Sigma-Aldrich, cat. no. L8000)

L-Lysine-HCl (Sigma-Aldrich, cat. no. L5501)

L-Methionine (Sigma-Aldrich, cat. no. M9625)

L-Phenylalanine (Sigma-Aldrich, cat. no. P2126)

L-Serine (Sigma-Aldrich, cat. no S4500)

L-Threonine (Sigma-Aldrich, cat. no T8625)

L-Tryptophan (Sigma-Aldrich, cat. no T0254)

L-Tyrosine (Sigma-Aldrich, cat. no T3754)

L-Valine (Sigma-Aldrich, cat. no V0500)

Yeast drop-out supplements for synthetic medium lacking histidine (Sigma-Aldrich, cat. no. Y-1751-20G)

SORB-His plates, SD-His plates, and TOP agar-His (Teknova, Inc.) (http://www.teknova.com)

Sorbitol (Sigma-Aldrich, cat. no. S1876-5 KG)

Polyethylene glycol 8000 (PEG) (Sigma-Aldrich, cat. no. 89510-1 KG-F)

14 M beta-mercaptoethanol (ME) (Sigma-Aldrich, cat. no. M3148-100 ML)

**CAUTION **It is highly toxic on contact with skin and is harmful if inhaled or swallowed.

Zymolyase 20T (MP Biomedical, cat. no. 08320921)

Sodium dodecyl sulfate (SDS) (Bio-Rad Laboratories, cat. no. 161-0301)

Tris hydroxymethyl-aminomethane (Bio-Rad Laboratories, cat. no. 161-0719)

Ethylenediaminetetraacetic acid (EDTA) (Sigma-Aldrich, cat. no. E-5134)

Diethylpyrocarbonate (DEPC) (Sigma-Aldrich, cat. no. D5758-50 ML)

Isopropanol (100%) (Sigma-Aldrich, cat. no. 19516-500 ML)

**CAUTION **It can cause eye and skin irritation.

Potassium acetate (KAc) (Sigma-Aldrich, cat. no. P1190-500G)

Sodium acetate (NaAc) (Sigma-Aldrich, cat. no. S2889-250G)

Sodium chloride (NaCl) (Sigma-Aldrich, cat. no. S7653-250G)

Potassium chloride (KCl) (Sigma-Aldrich, cat. no. P9333-500G)

Sodium hydroxide (NaOH) (Sigma-Aldrich, cat. no. S8045-500G)

Ethidium bromide (EtBr) (Sigma-Aldrich, cat. no. E7637-1G)

**CAUTION **It is highly toxic on contact with skin.

Glacial acetic acid (Sigma-Aldrich, cat. no. A9967-500G)

**CAUTION **It is a toxic and corrosive solution. It can cause irritation on contact with skin and is harmful if inhaled or swallowed.

Chloramphenical (Sigma-Aldrich, cat. no. C0378-5G)

Ethyl alcohol (EtOH) (Sigma-Aldrich, cat. no. E3884-1G)

Phenol:Chloroform:Isoamyl Alcohol mixture (Sigma-Aldrich, cat. no. 77617-100 ML)

**CAUTION **It is highly toxic on contact with skin and if swallowed.

QIAGEN Plasmid Maxi Kit (QIAGEN, cat. no. 12162)

QIAquick Gel Extraction Kit (QIAGEN, cat. no. 28704)

Low Range PFG marker (New England Biolabs, cat. no. N0350S)

PFGE standard, 8.3-48.5 kb (Bio-Rad Laboratories, cat. no. 170-3707)

MassRuler™ DNA Ladder, Low Range (Fermentas, cat. no. SM0383)

MassRuler™ DNA Ladder, High Range (Fermentas, cat. no. SM0393)

UltraPure ™ Agarose (Invitrogen, cat. no. 15510-0270)

Pulsed field certified agarose (Bio-Rad Laboratories, cat. no. 162-0137)

T4 DNA Ligase (New England Biolabs, cat. no. M0202S)

Phi29 DNA polymerase (New England Biolabs, cat. no. M0269L)

Phi29 Random Hexamer Primers (Fidelity systems, cat. no. R106)

### Equipment

100 × 13 mm sterile disposable Petri plates (USA Scientific, Inc., cat. no. 8609-5010)

Corning 500 ml Vacuum Filter/Storage Bottle System, 0.22 μm, Cellulose Acetate Membrane (Corning Life Sciences, cat. no. 430769)

Pyrex, Baffled Culture Flasks (Fisher Scientific Ltd., cat. no. 10-041-5B). This flask is sterilized and used to grow the 50 ml YEPD yeast culture for transformation.

Inoculating loop (Nichrom wire) (Fisher Scientific Ltd., cat. no. 13-104-5). This is used for streaking and inoculating cultures.

Humboldt Bunsen Burner (Fisher Scientific Ltd., cat. no. 22-043-141). This is used to sterilize the inoculating loop.

Fisherbrand premium microcentrifuge 2.0 ml tubes (Fisher Scientific Ltd., cat. no. 05-408-138*)*. Tubes are autoclaved to sterilize. These tubes are used for mixing spheroplasts with different transformation reagents.

Corning 15 ml polypropylene sterile screw-cap disposable graduated centrifuge tubes (Fisher Scientific Ltd., cat. no. 05-538-51). These tubes are used to mix yeast spheroplasts with melted SRB-TOP-His agar medium, which is then poured onto the plates with SORB-His regenerative agar.

Corning 50 ml sterile screw-cap disposable graduated centrifuge tubes (Fisher Scientific Ltd., cat. no. 05-538-55). These tubes are used to collect the yeast cells and spheroplasts during transformation.

New Brunswick Incubating 12500 Shaker (Fisher Scientific Ltd., cat. no. 14-728-3). This is used to grow liquid yeast cultures.

Microcentrifuge (Fisher Scientific Ltd., Eppendorf Model 5417R, cat. no. 05-406-8A). This is used to pellet the yeast cells in 2.0 ml microcentrifuge tubes.

Centrifuge (Thermo Scientific, Sorvall Legend RT Plus, Benchtop Centrifuge, cat. no. 75004377). This is used to pellet the yeast cells in 50 ml centrifuge tubes.

Rotor (Thermo Scientific, TTH-750 High-Capacity Swing-Out Rotor, cat. no. 75006445). Rotor for Sorvall centrifuge.

Forced-Air Incubator (Fisher Scientific Ltd., cat. no. 11-690637F). This is set to 30°C for growth of plate cultures.

Thermo Precision General-Purpose Water Baths for 50°C and 70°C (Fisher Scientific Ltd., cat. no. 15-460-2). The 50°C bath is used to keep SORB-TOP-agar medium melted, while the 70°C bath is used during yeast DNA isolation.

Sterile flat toothpicks. They may be purchased from a local grocery store. Place wide end down into a 100 ml beaker, cap with aluminum foil, and then autoclave. They can then be used by turning the beaker on its side and removing one at a time

Spectrophotometer (Fisher Scientific Ltd., cat. no. S42475P). This is used at 660 nm for OD in order to determine yeast cell numbers in cultures and used with plastic cuvettes (Fisher Scientific Ltd., cat. no. 14-385-938).

Gene Pulser Xcell Total System (Bio-Rad Laboratories, cat. no. 165-2660). This is used to transform yeast recombinant molecules from yeast cells into *E. coli *cells.

Pulser Cuvette (Bio-Rad Laboratories, cat. no. 165-2086). This is used for electroporation experiments.

CHEF Mapper XA Chiller System (Bio-Rad Laboratories, cat. no. 170-3670). This is used to check the size of inserts within BAC samples.

Gel-Doc 2000 System (Bio-Rad Laboratories, cat. no. 12621-084)

Savant SPD131DDA SpeedVac (Thermo Scientific, cat. no. SPD131DDA-115)

### Reagent setup

#### The basic TAR cloning vector

The basic pNK-TAR vector contains a yeast selectable marker (*HIS3*), a yeast origin of replication *ARSH4*, a yeast centromeric sequence *CEN6 *from yeast chromosome VI, a BAC cassette with a bacterial selectable marker that allows the YAC clones to be transferred into *E. coli *cells, and a mammalian selectable marker, the *Neo *or *BS *gene. The vector contains targeting sequences (or hooks) homologous to a repeat of interest that are inserted into the polylinker. The amount of the linearized TAR vector needed for spheroplasts transformations is ~100 ng diluted in 20-50 μl of water (keep at -20°C). The vector and its more detailed description are available upon request.

#### Sorbitol solution (1 M)

Add 182 g of sorbitol to about 700 ml of distilled/deionized water in a 1000 ml beaker. Stir until dissolved. Make the volume up to 1000 ml in a 1000 ml measuring cylinder and mix thoroughly. The solution is filter sterilized. Sorbitol solution can be stored at room temperature (RT).

#### SPE solution

(1M Sorbitol, 10 mM Na2EDTA, 0.01 M Na phosphate, pH 7.5). Add 91 g of sorbitol, 1.04 g of Na2HPO_4 _× 7H_2_O, 0.16 g of NaH_2_PO_4 _× 1H_2_O, and 10 ml of 0.5M EDTA, pH 7.5 to about 400 ml of distilled/deionized water in a 500 ml beaker. Stir until dissolved. Make the volume up to 500 ml in a 500 ml measuring cylinder and mix thoroughly. The solution is filter sterilized. SPE solution can be stored at RT.

#### SOS solution

(1M Sorbitol, 6.5 mM CaCl2, 0.25% yeast extract, 0.5% peptone). Add 91 g of sorbitol, 1.25 g of bacto yeast extract, 2.5 g of bacto peptone, and 3 ml of 1M CaCl_2 _to about 400 ml of distilled/deionized water in a 500 ml beaker. Stir until dissolved. Make the volume up to 500 ml in a 500 ml measuring cylinder and mix thoroughly. The solution is filter sterilized. SOS solution can be stored at RT.

#### STC solution

(1M Sorbitol, 10 mM CaCl2, 10 mM Tris-HCl, pH 7.5). Add 91 g of sorbitol, 5 ml of 1M Tris-HCl pH 7.5 and 5 ml of 1M CaCl_2 _to about 400 ml of distilled/deionized water in a 500 ml beaker. Stir until dissolved. Make the volume up to 500 ml in a 500 ml measuring cylinder and mix thoroughly. The solution is filter sterilized. STC solution can be stored at RT.

#### Zymolyase solution

(10 mg/ml of zymolyase 20T in 25% glycerol). Add 200 mg of zymolyase 20T and 1 ml of Tris-HCl pH 7.5 to 9 ml of distilled/deionized water and 10 ml of 50% glycerol. Stir until dissolved. Keep as frozen aliquots of 500 ml at -20°C.

#### PEG MW 8000 solution

(20% (w/v) polyethylene glycol 8000, 10 mM CaCl2, 10 mM Tris-HCl, pH 7.5). Add 20 g of PEG 8000, 1 ml of 1M Tris-HCl pH 7.5 and 1 ml of 1M CaCl_2 _to about 70 ml of distilled/deionized water in a 150 ml beaker. Stir until dissolved. Use a hot plate to gently warm the solution if necessary. Make the volume up to 100 ml in a 100 ml measuring cylinder and mix thoroughly. The solution is filter sterilized. Keep the PEG solution at RT. Make a new PEG solution every month to avoid reduction of the yield of transformants.

#### SDS solution (2%)

Add 2.0 g of sodium dodecyl sulfate to 80 ml of distilled/deionized water in a 100 ml beaker. Stir until dissolved. Make the volume up to 100 ml in a 100 ml measuring cylinder. SDS solution can be stored at RT.

#### SDS solution (10%)

Add 10.0 g of sodium dodecyl sulfate to 80 ml of distilled/deionized water in a 100 ml beaker. Stir until dissolved. Make the volume up to 100 ml in a 100 ml measuring cylinder. SDS solution can be stored at RT.

#### Ethidium Bromide (EtBr) (10 mg/ml)

Add 1 g of ethidium bromide to 100 ml of distilled/deionized water. Stir well for several hours to ensure that the dye has dissolved. Wrap the container in aluminum foil or transfer the solution to a dark bottle and store at RT.

#### KAc solution (5 M)

Dissolve 29.5 g of solid KAc (potassium acetate) in 75 ml of distilled/deionized water and add 11.5 ml of glacial acetic acid. Adjust volume to 100 ml with distilled/deionized water and filter-sterilize. Store at RT.

#### NaAc solution (3M, pH 5.2)

Dissolve 40.8 g of solid NaAc (sodium acetate/3H_2_0) in 80 ml of distilled/deionized water. Adjust the pH to 5.2 with glacial acetic acid. Adjust the volume to 100 ml with distilled/deionized water. Store at RT.

#### EDTA solution (0.5 M, pH 7.5)

Add 186.1 g of disodium EDTA × 2H_2_O to 800 ml of distilled/deionized water. Stir vigorously on a magnetic stirrer. Adjust pH to 7.5 with 1.0 N NaOH. The disodium salt of EDTA will not go into solution until the pH of the solution is adjusted to ~ 8.0 by the addition of 1.0 N NaOH. Sterilize by autoclaving. EDTA solution can be stored at RT.

#### Tris-HCl solution (1.0 M, pH 7.5)

Dissolve 121.1 g of Tris base in 800 ml of distilled/deionized water. Add 70 ml of concentrated HCl to adjust the pH to 7.5. Make the volume up to 1000 ml in a 1000 ml measuring cylinder and mix thoroughly. Tris-HCl solution can be stored at RT.

#### EDTA mix

(50 mM EDTA, 0.2% SDS). Mix 1 ml of 0.5 M EDTA solution, pH 7.5 and 20 ml of 10% SDS solution with 79 ml of distilled/deionized water. EDTA mix solution can be stored at RT.

#### RNase A (10 mg/ml)

Make 10 times dilution from 100 mg/ml RNase A stock (provided by either Qiagen Large-Construct Kit or Qiagen DNA miniprep Kit) with sterile water. The solution can be stored for up to 6 months at RT.

#### Sodium chloride solution (5 M NaCl)

Dissolve 5.8 g of solid NaCl (sodium chloride) in 100 ml of distilled/deionized water. Stir until dissolved. The solution can be filter sterilized. Store at RT.

#### Sodium hydroxide (1M NaOH)

Dissolve 40 g of solid NaOH (sodium hydroxide) in 100 ml of distilled/deionized water. Stir until dissolved. Store at RT.

#### Potassium chloride solution (5 M KCl)

Dissolve 7.4 g of solid KCl (potassium chloride) in 100 ml of distilled/deionized water. Stir until dissolved. The solution can be filter sterilized. Store at RT.

#### SOC solution

(2% Bacto tryptone, 0.5% Bacto yeast extract, 10 mM NaCl, 2.5 mM KCl). Add 2 g of bacto tryptone, 0.5 g of bacto yeast extract, 200 ml of 5M NaCl, and 50 ml of 5M KCl to about 100 ml of distilled/deionized water in a 250 ml beaker. Stir until dissolved. Make the volume up to 100 ml in a 100 ml measuring cylinder and mix thoroughly. Transfer the solution to a glass storage bottle and autoclave for 30 min. Alternatively, the solution can be filter sterilized. SOS solution can be stored at RT.

#### Chloramphenical solution (12 mg/ml in EtOH)

It is used as a selection agent for transformed cells containing chloramphenicol resistance genes. Add 1.2 g of chloramphenical to 100 ml of ethanol (EtOH) in a 250 ml beaker. Stir until dissolved. Transfer the solution into a glass storage bottle. Chloramphenical solution can be stored at -20°C.

#### YEPD liquid medium

(2% D-glucose, 1% Bacto yeast extract, 2% Bacto peptone, 2% Bacto agar, adenine hemisulfate 20 mg l^-1^). Add 20 g of D-glucose, 20 g of Bacto peptone, 10 g of Bacto yeast extract, and 5 ml of adenine solution (4 mg/ml) to about 1000 ml of distilled/deionized water in a 1000 ml beaker. Stir until dissolved. Make the volume up to 1000 ml in a 1000 ml measuring cylinder and mix thoroughly. Transfer the solution into a glass storage bottle and autoclave for 21 min. It can be stored at RT.

#### YEPD medium with agar

(2% D-glucose, 1% Bacto yeast extract, 2% Bacto peptone, 2% Bacto agar, adenine hemisulfate 20 mg l^-1^). Add 20 g of D-glucose, 20 g of Bacto peptone, 10 g of Bacto yeast extract, and 5 ml of adenine solution (4 mg/ml) to about 1000 ml of distilled/deionized water in a 1000 ml beaker. Stir until dissolved. Make the volume up to 1000 ml in a 1000 ml measuring cylinder and mix thoroughly. Transfer the solution into a 2000 ml glass flask, add 20 g of Bacto agar, and autoclave for 30 min. It can be stored at RT. Alternatively, YEPD medium can be purchased from Teknova, Inc. (http://www.teknova.com).

#### LB liquid medium

(1% Bacto tryptone, 0.5% Bacto yeast extract, 1% NaCl, 2 mM NaOH, pH 7.4). Add 10 g of Bacto tryptone, 5 g of Bacto yeast extract, 10 g of NaCl, and 200 ml of 1M NaOH to about 1000 ml of distilled/deionized water in a 1000 ml beaker. Stir until dissolved. Make the volume up to 1000 ml in a 1000 ml measuring cylinder and mix thoroughly. Transfer the solution to a glass storage bottle and autoclave for 15 min. Alternatively, the solution can be filter sterilized. The solution can be stored at RT.

#### LB medium with agar

(1% Bacto tryptone, 0.5% Bacto yeast extract, 1% NaCl, 2% agar, 2 mM NaOH, pH 7.4 ). Add 10 g of Bacto tryptone, 5 g of Bacto yeast extract, 10 g of NaCl, 20 g of agar, and 200 ml of 1M NaOH to about 1000 ml of distilled/deionized water in a 1000 ml beaker. Stir until dissolved. Make the volume up to 1000 ml in a 1000 ml measuring cylinder and mix thoroughly. Transfer the solution to a glass storage bottle and autoclave for 30 min. It can be stored at RT.

#### LB-Cm liquid medium

(12.5 mg/ml chloramphenical). Add 1 ml of chloramphenical solution to 1000 ml of LB medium. Stir well. It can be stored at +4C° for at least six months.

#### LB-Cm plates

(12.5 mg/ml chloramphenical). Add 1 ml of chloramphenical solution to 1000 ml of LB medium with agar. Mix well. Pour approximately 20 ml into each Petri dish. The plates can be stored at +4C° for at least six months.

#### Selection medium SORB-TOP-His

(without histidine) 1 M Sorbitol, 2% D-glucose, 0.17% Yeast Nitrogen Base, 0.5% (NH4)2SO4, 3% Bacto agar containing the following supplements: 0.006% adenine sulfate, 0.006% uracil, 0.005% L-arginine HCl, 0.008% L-aspartic acid, 0.01% L-glutamic acid, 0.005% L-isoleucine, 0.01% L-leucine, 0.012% L-lysine. HCl, 0.002% L-methionine, 0.005% L-phenylalanine, 0.0375% L-serine, 0.01% L-threonine, 0.005% L-tryptophan, 0.005% L-tyrosine, 0.015% L-valine. Alternatively, synthetic complete medium can be purchased from Teknova, Inc. (http://www.teknova.com).

#### Petri plates with selection medium SORB-His

(without histidine) 1 M Sorbitol, 2% D-glucose, 0.17% Yeast Nitrogen Base, 0.5% (NH4)2SO4, 2% Bacto agar supplemented as described for SORB-TOP-His. The medium is mixed with double-distilled water and then adjusted to pH 5.6 with 1.0 N NaOH and autoclaved. Alternatively, plates with synthetic complete medium can be purchased from Teknova, Inc. (http://www.teknova.com).

#### Petri plates with selection medium SD-His

(without histidine) 2% D-glucose, 0.17% Yeast Nitrogen Base, 0.5% (NH4)2SO4, 2% Bacto agar supplemented as described for SORB-TOP-His. The medium is mixed with double-distilled water and then adjusted to pH 5.6 with 1.0 N NaOH and autoclaved. Alternatively, plates with synthetic complete medium can be purchased from Teknova, Inc. (http://www.teknova.com).

### Equipment setup

#### Agarose gel electrophoresis

DNA is separated on a 1% (wt/vol) or 2% agarose/1XTBE electrophoresis gel by applying 4.5 V cm^-1 ^for 3 h. After electrophoresis, the gel is stained with EtBr (1/10,000 dilution) for 15-30 min and detected by the Gel Doc System.

#### Clamped homogeneous electrical field (CHEF) electrophoresis

CHEF analysis is performed in a 1% (wt/vol) pulsed field certified agarose gel with 0.5XTBE buffer circulated through a cooler set at 14°C. Typical forward parameters are as follows: 9.0 V/cm, initial switch 0.11 s, and final switch 0.92 s, with linear ramp. Typical reverse parameters are: 6.0 V/cm, initial switch 0.11 s, final switch 0.92 s, with linear ramp for 20.5 h. After electrophoresis, the gel is stained with EtBr (1/10,000 dilution) for 15-30 min and detected by the Gel-Doc System.

#### Electroporation into *E. coli *cells

Electroporation is performed with DH10B competent cells using a Bio-Rad Gene Pulser MXcell Electroporation System with the settings 2.5 kV, 200 W, and 25 μF.

### Procedure

#### Preparation of the circular DNA template for rolling circle amplification (RCA) TIMING 18 hr

**Figure 1:**
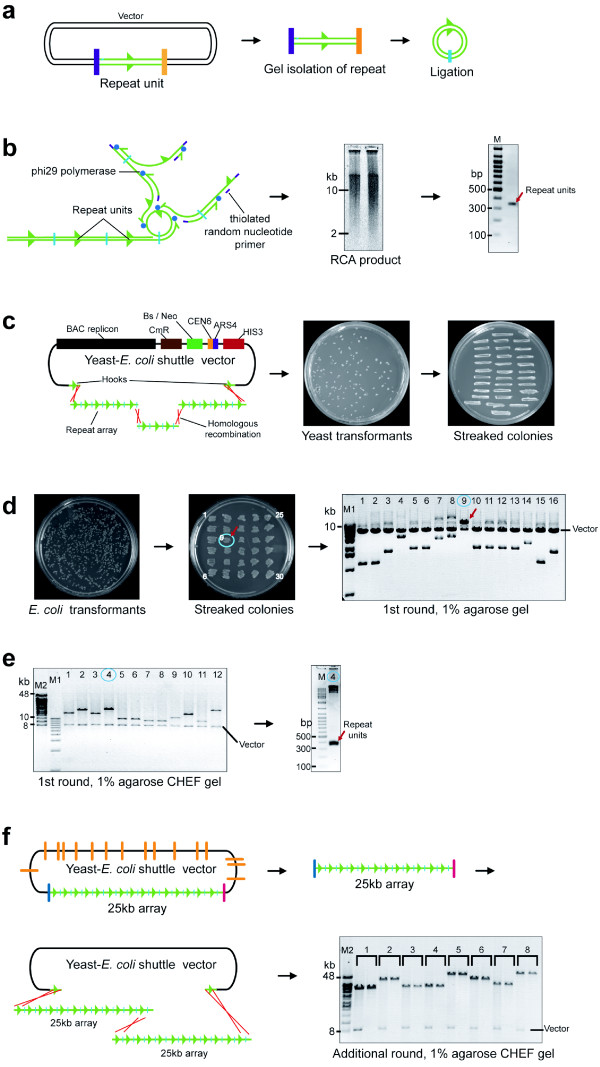
**Scheme of the repeats assembly into synthetic DNA arrays**. (**a**) The DNA repeat isolated from the vector (in purple and orange) is ligated to make a circular molecule. (**b**) The RCA products were generated from a 340 bp alphoid dimer. Cleavage of RCA products with an enzyme results in restoration of the input repeat. (**c**) Recombinational assembly includes co-transformation of RCA products into yeast along with a TAR vector (YAC/BAC) containing repeat-specific targeting hooks. End-to-end recombination of DNA fragments, followed by interaction of the recombined fragments with the vector hooks, results in the rescue of arrays as circular YACs. His^+ ^transformants and pooled colonies are shown. (**d**) Transferring of YACs into bacterial cells. *E. coli *transformants and streaked colonies are shown. BAC DNAs from randomly picked up colonies were restricted by an endonuclease that releases the vector part (7 kb) and arrays. The size of arrays varies from 2 to 12 kb. BAC DNA from colony #9 with the largest array is marked by the red arrow. (**e**) CHEF analysis of BACs with the largest arrays chosen after screening 60 *E. coli *transformants. The size of the inserts varies from 9 to 25 kb. The tandem repeat structure of one array (clone #4 with the size ~25 kb) is confirmed by EcoRI digestion (**f**) An additional round of recombinational assembly to further increase the size of the array. Representative CHEF analysis of 8 BACs is shown. Restriction of BAC DNAs was done by an endonuclease that cleaves the molecule at insert/vector junctions (arrays are between 40 and 60 kb) and by double digestion with an additional endonuclease that cuts the vector part completely.

Before the RCA reaction, the repeat should be cloned into TOPO vector and sequenced. The sequenced repeat is isolated from the vector DNA using an appropriate endonuclease that cleaves at insert/vector junctions (see Figure 1a).

1. Mix 40 μl (~10 μg) of vector DNA with 15 μl of 10X buffer, 10 μl (100 units) of enzyme, and 85 μl of water. Total volume of the reaction is 150 μl. Keep at 37°C for 2 hr.

2. Load the restriction mixture into the wells of 2% w/v agarose/EtBr gel.

3. Run the gel at 4.5 V cm^-1 ^for 1 hr.

4. Cut the agarose slice containing the fragment from the gel.

5. Isolate the DNA fragments by QIAquick Gel Extraction Kit. Final volume is ~70 μl.

**CRITICAL STEP **Take 3 μl from Step 5, load into the well of 2% agarose/EtBr gel, and run the gel to ensure that the DNA was not lost during isolation.

6. Take 60 μl from Step 5 and mix with 30 μl of x10 DNA ligase buffer, 10 μl of DNA ligase, and 200 μl of water. Total volume of the ligation reaction is 300 μl. Incubate the reaction overnight at 16°C.

**CRITICAL STEP **The ligation reaction should be performed with diluted DNA to prevent inter-molecular ligation. To ensure that the ligation reaction has worked, take 3 μl of the isolated fragment before the ligation reaction (from Step 5) and 30 μl of the ligation mixture after overnight incubation (from Step 6). Load into the wells of 1% agarose gel, run the gel, and stain the gel by ethidium bromide. The ligated fragment moves slower than the initial isolated fragment. **CAUTION **Ethidium bromide is highly toxic on contact with skin.

7. Load the ligation mixture (from Step 6) into the wells of 1% agarose/EtBr gel. Run for 1-2 hr. Cut off the circular supercoiled DNA from the gel. Isolate the DNA by QIAquick Gel Extraction Kit. Final volume is 150 μl (~1.5 μg DNA) (see Figure 1a).

#### DNA repeat amplification reaction by RCA TIMING 13 hr (see Figure 1b)

8. Denaturize 5 μl of template DNA (~50 ng) (from Step 7) by adding 5 μl of 10 mM NaOH. Incubate at RT for 5 min.

9. Mix 2.0 μl (~10 ng) of the denaturized DNA with 2.5 μl (25 units) of Phi29 DNA Polymerase (10 units/μl), 40 μl of 2.5 μM dNTPs, 5 μl of 500 μM Phi29 Random Hexamer Primer, 10 μl of 2X annealing buffer, and 40.5 μl of deionized water. Total volume of the reaction is 100 μl.

10. Mix well by pipetting. Spin the sample briefly before placing in the thermocycler.

11. Incubate reaction at 30°C for 12 hr.

**CRITICAL STEP **Take 12 μl of the overnight RCA reaction mixture for analysis. Digest 6 μl with an endonuclease that cleaves the RCA products into its original repeat unit. Leave the other 6 μl undigested. Use 1% agarose/EtBr gel to see the RCA reaction products and 2% agarose/EtBr gel to see the repeat unit (see Figure 1b).

12. Heat inactivate Phi29 DNA polymerase by incubation at 65°C for 10 min.

13. Precipitate the RCA products with 2.5 volume of EtOH.

14. Pellet the precipitate by centrifugation for 5 min at maximum Eppendorf minicentrifuge speed (20.000 × g).

15. Remove the supernatant and wash the pellet with 70% EtOH.

16. Resuspend the damp DNA pellet in 100 μl of water.

**PAUSE POINT **The RCA product may be kept at 4°C for several weeks.

#### Recombinational cloning of RCA products using a TAR vector in yeast (see Figure 1c)

##### Preparation of the yeast culture TIMING overnight

17. One day before the TAR cloning experiment, inoculate three different size colonies of the host yeast strain VL6-48 freshly grown on a YEPD plate in three separate 50 ml aliquots of YEPD medium in three 250-ml Erlenmeyer flasks. Grow the cultures overnight at 30°C with vigorous shaking to assure good aeration.

#### Preparation of competent yeast spheroplasts TIMING 2-3 hr

18. In the morning, measure the optical density (OD) of the cultures with 20 min intervals until an OD_660 _of ~2.0 is achieved in the flask.

**CRITICAL STEP **For actual measurement, dilute the culture 1/10 in water; the density should be between 0.18-0.22. The culture with such an optical density is ready for the preparation of highly competent spheroplasts. This optical density corresponds to approximately 2 × 10^7 ^cells per ml.

19. Transfer the yeast culture into a 50-ml corning tube and pellet the cells by centrifugation for 5 min at 1,000 × g, 5°C. Remove and discard the supernatant.

20. Resuspend the cell pellet in 30 ml of sterile water by vortexing and centrifuge for 5 min at 3,000 × g, 5°C. Remove and discard the supernatant.

21. Resuspend the cell pellet in 20 ml of 1 M Sorbitol solution by vortexing and centrifuge for 5 min at 3,000 × g, 5°C. Remove and discard the supernatant.

**PAUSE POINT **Yeast cells in 1 M Sorbitol solution may be kept at 4°C overnight.

22. Resuspend the cell pellet in 20 ml of SPE solution. Add 40 μl of Zymolyase solution and 40 μl of ME into the tube, mix wel,l and incubate at 30°C for ~ 20-40 min with a slow shaking. (Note that the treatment time varies depending on the zymolyase stock).

23. Check the level of spheroplasting by comparison of theoptical densities of the cell suspension in 1 M Sorbitol solution versus 2% SDS solution every 20 min.

**CRITICAL STEP **To measure the OD_660 _difference, 200 μl aliquots of the zymolyase-treated cell suspension are diluted 10-fold by 1 M Sorbitol solution and 2% SDS solution. The spheroplasts are determined to be ready when the difference between the two OD_660 _readings is 3- to 5- fold. Both underexposure and overexposure to zymolyase greatly affects transformation efficiency. From this point on, extreme care must be taken to avoid lysing the delicate spheroplasts: very slow, gentle resuspensions are necessary.

24. Centrifuge the spheroplasts for 10 min at 570 × g, 5°C. Decant the supernatant, add 50 ml of 1.0 M Sorbitol solution, then rock very gently to resuspend the pellet. Pellet the spheroplasts again by centrifugation for 10 min at 300-600 × g, 5°C.

25. Repeat the wash with 50 ml of 1 M Sorbitol solution one more time and gently resuspend the final pellet in 2.0 ml of STC solution.

**PAUSE POINT **The spheroplasts are ready for transformation and are stable at RT for at least one hour.

#### Transformation of spheroplasts by RCA DNA products along with a TAR vector TIMING 2.0-2.5 hr

26. Mix gently 200 μl of spheroplast suspension with 2-4 μg of RCA DNA products and 10-40 ng of the linearized TAR vector in a 2.0 ml Eppendorf tube. Incubate for 10 min at RT. (Therefore, the total number of samples may be 10 for 2.0 ml of spheroplasts in STC solution obtained from 50 ml of the original culture).

27. Add 800 μl of PEG 8000 solution into each 2 ml Eppendorf tube. Gently mix by inverting and incubate for 10 min at RT.

28. Pellet the spheroplasts by centrifugation in the Eppendorf microfuge for 5 min at 300-500 × g, 5°C. Remove the supernatant and gently resuspend the spheroplasts in each tube with 800 μl of SOS solution using a Pipetman.

29. Incubate the spheroplasts for 40 min at 30°C without shaking.

30. Transfer the spheroplasts from each tube into a 15-ml corning tube containing 7.0 ml of melted SORB-TOP-His medium (equilibrated at 55°C) using a Pipetman. Gently mix and quickly pour agar onto SORB-His plates with selective medium (without histidine) containing 1 M Sorbitol.

**PAUSE POINT **Keep the plates at 30°C for 5 days until the transformants become visible (see Figure 1c).

### Organizing transformants into pools TIMING overnight

31. Transfer ~200-600 primary transformants using toothpicks onto SD-His plates lacking histidine (streak ~30-50 transformants per each plate to make pools).

32. Incubate the plates with pools of transformants at 30°C overnight (see Figure 1c).

#### DNA isolation from yeast transformants TIMING 5 hr

33. Wash the yeast cells out from each plate with 5 ml water into a 50-ml corning tube and pellet the cells by centrifugation for 5 min at 1,000 × g, 5°C. Remove and discard the supernatant.

34. Resuspend the cell pellet in 5 ml of 1 M Sorbitol solution by vortexing and then transfer 1 ml of the suspension into a 2.0 ml Eppendorf microfuge tube and spin for 30 sec. Remove and discard the supernatant.

35. Resuspend the cell pellet in 0.5 ml of SPE solution containing ME (1/1000 dilution). Add 40 μl of Zymolyase solution and incubate for 1 hr at 37°C.

36. Harvest the spheroplasts by centrifugation for 5 min at 3,000 × g in the Eppendorf microfuge and resuspend the pellet in 0.5 ml of EDTA mix with a Pipetman.

37. Add 50 μl of 2% SDS and mix with a Pipetman.

38. Lyse the spheroplasts completely by incubation at 70°C for 15 min.

39. Add 50 μl of 5 M KAc solution into the tube, mix well, and let the tube sit on ice for 30 min.

40. Pellet the precipitate by centrifugation for 15 min at maximum Eppendorf minifuge speed (20,000 × g).

41. Transfer the supernatant into a fresh microcentrifuge tube, then add an equal volume of RT isopropanol, mix well, and pellet the DNA by centrifugation for 5 min at maximum Eppendorf minifuge speed (20,000 × g). Remove the supernatant as much as possible and dry the tube by inverting on blotting paper. **CAUTION **Isopropanol can cause eye and skin irritation.

41. Rinse the pellet with 70% ethanol and then resuspend the damp DNA pellet in 0.3 ml of water.

**PAUSE POINT **Samples can be incubated at 4°C overnight to complete dissolving the DNA. The DNA samples can be left frozen for up to several months at -20°C.

#### Electroporation of yeast DNA into *E. coli *cells TIMING 1.5 hr (see Figure 1d)

42. Take 1 μl of DNA isolated from yeast transformants (from Step 41) to electroporate 20 μl of the *E. coli *DH10B competent cells using a Bio-Rad Gene Pulser with the settings 2.5 kV, 200 W, and 25 μF.

43. After electroporation, add 1 ml of SOC solution to a cuvette containing the electroporated cells, mix well by pipetting up and down, and transfer into a microcentrifuge tube.

44. Incubate the cells for 1 hr at 30°C.

45. Spread 10 μl, 50 μl, and 100 μl of the cell suspension onto three separate LB-Cm plates.

46. Incubate the plates for two days at 30°C (see Figure 1d).

**CRITICAL STEP **Growing of *E. coli *transformants at 30°C rather than 37°C is needed to keep the integrity of the assembled tandem repeats array.

#### Miniprep BAC DNA purification from *E. coli *transformants TIMING 3 hr

47. Inoculate 30-60 15 ml tubes containing 2 ml of LB-Cm liquid medium with clearly isolated colonies of *E. coli *Cm^R ^transformants. Incubate cultures overnight in a shaking incubator set at 30°C and 150 rpm.

**CRITICAL STEP **Choose small size *E. coli *transformant colonies. It has been noted that large arrays are preferentially found amongst smaller colonies. When setting up the inoculation, use a toothpick to first pick up a transformant, then streak it onto LB-Cm plates before dropping that toothpick into the 2 ml culture medium. Streaked transformants are grown at 30°C and used for further analysis when needed (see Figure 1d).

48. Centrifuge the tubes at 3,400 × g for 10 min to pellet the cells. Discard spent media using an aspirator.

49. Resuspend the cell pellet in 200 μl of Buffer P1 without RNAse A and transfer the cell suspension into a 1.5 ml micro-centrifuge tube.

50. Add 200 μl of Buffer P2 and mix thoroughly by inverting the tubes 3-4 times.

51. Add 200 μl of Buffer P3 and mix thoroughly by inverting the tubes 5-6 times.

52. Centrifuge the tubes for 15 min at 20,000 × g, 4°C in a micro-centrifuge. A compact white pellet will form.

53. Transfer the supernatant into a fresh 1.5 ml micro-centrifuge tube.

54. Add an equal amount of isopropanol and mix thoroughly by inverting the tubes 5-6 times.

55. Pellet the precipitate by centrifugation for 15 min at 20,000 × g, 4°C. A light white pellet will form.

56. Dissolve the pellet in 300 μl of water.

57. Add 300 μl of phenol:chlorophorm:isoamyl alcohol (25:24:1), pH 8.0 and mix thoroughly by inverting the tubes 10 times. **CAUTION **It is highly toxic on contact with skin and if swallowed.

58. Centrifuge the tubes for 5 min at 20.000 × g, 4°C in a micro-centrifuge.

59. Transfer the upper phase supernatant into a fresh 1.5 ml micro-centrifuge tube.

60. Add 1/10 of volume of 3 M NaAc (30 μl) and an equal amount of isopropanol (300 μl). Mix thoroughly by inverting the tubes 5-6 times. **CAUTION **Isopropanol can cause eye and skin irritation.

61. Pellet the precipitate by centrifugation for 15 min at 20.000 × g, 4°C in a micro-centrifuge. A small white pellet will form.

62. Dissolve the pellet in 30 μl of water with 10 μg/ml RNAse A.

#### Checking the size of BAC DNA repeat arrays TIMING 24 hr

63. Ascertain the size of each DNA repeat array by cutting it out of the BAC vector using the appropriate restriction enzyme(s). Digest 20 μl of the sample from Step 62 in a total volume of 40 μl for 2 hours.

64. Take half of the sample from Step 63 (20 μl) and run the DNA digest using gel electrophoresis with a 1.0% 1xTBE agarose gel, 1xTBE buffer, and a constant voltage setting of 6V/cm for approximately 2 hours.

65. Stain the gel with ethidium bromide for 10 min and photograph the DNA bands using a Gel-Doc 2000 system. Typical results are shown in Figure 1d.

66. Choose several BACs with the slowest band mobility and thus carrying the largest DNA array for further analysis. The actual size of the BAC inserts is determined by CHEF gel electrophoresis. Run the remaining half of the digest sample from Step 63 (20 μl) on a CHEF Mapper XA Chiller System.

67. Stain the gel with ethidium bromide for 15-30 min and photograph the DNA bands using a Gel-Doc System. Typical results are shown in Figure 1e. **CAUTION **Ethidium bromide is highly toxic on contact with skin.

68. Choose the BAC with the biggest DNA array. Check the integrity of the amplified repeat by digesting the remaining 10 μl sample from Step 62 with an endonuclease that cleaves the array into its original repeat unit (see Figure 1e).

#### Successive increase of the DNA array length (optional) (see Figure 1f)

In situations when the size of the array needs to be amplified further, a second round of recombinational cloning is carried out. To do this, make a large-scale BAC DNA purification from the sample with the largest array first (Steps 69-82), then followed by another round of recombinational cloning (Steps 83-89).

#### QIAGEN large-scale BAC DNA purification TIMIMG 3 hr

69. Inoculate *E. coli *culture of the chosen sample (from Steps 66-68) containing the largest DNA array into a 250-ml Erlenmeyer flask with 200 ml of LB-Cm liquid medium. Incubate overnight at 30°C with moderate shaking (150 rpm).

**CRITICAL STEP **Stability of the assembled tandem repeat array is maintained by growing *E. coli *transformants at 30°C rather than 37°C. Also for this reason, *E. coli *cultures are grown with minimal aeration, i.e., a large culture volume (200 ml) in a relatively small flat bottom flask (250-500 ml) and with shaking at 150 rpm. It has been noted that growth under conditions of good aeration induces structural instability of tandem repeats array.

70. Harvest the cells by centrifugation at 6.000 × g for 20 min at 4°C.

71. Resuspend the pellet in 10 ml of Buffer P1. Ensure that RNAse A has been added to Buffer P1.

72. Transfer the suspension into a sterile Corning 50-ml screw-cap disposable graduated centrifuge tube.

73. Add 10 ml of Buffer P2, mix gently by inverting 10 times, and incubate at RT for 5 min.

74. Add 10 ml of chilled Buffer P3, mix well by inverting 10 times and incubate on ice for 15 min.

75. Centrifuge at 4.000 × g for 30 min at 4°C.

76. Transfer the supernatant into a new 50-ml Corning tube by filtering the lysate through a double gauze or a folded filter pre-wetted with distilled water.

77. Equilibrate a QIAGEN-tip 500 column by applying 10 ml of Buffer QBT and allow the column to empty by gravity flow. This should be done immediately before applying the sample.

78. Apply the DNA sample from Step 76 to the QIAGEN column and allow it to enter the resin by gravity flow.

79. Wash the column twice with 30 ml of Buffer QC and allow it run through the column by gravity flow.

80. Elute the DNA with 15 ml of Buffer QF (pre-warmed to 65°C). Collect the eluate in a 50-ml Corning tube.

81. Precipitate the DNA by adding 11 ml of RT isopropanol to the eluted DNA. Mix and centrifuge immediately at 15.000 × g for 30 min at 4°C. Carefully decant the supernatant.

82. Air-dry the pellets for 10 min. Dissolve the DNA pellet in ~100 μl of water. The final concentration of BAC DNA will be ~100-200 ng/μl.

#### Second round of recombinational cloning

83. Digest 3-5 μg of BAC DNA containing the largest insert with the endonuclease that cleaves it at insert/vector junctions.

84. Eliminate TAR vector DNA with an additional restriction enzyme that selectively cuts the vector into very small fragments but leaves the array undigested.

85. Precipitate the final double digest with 1/10 volume of 3 M NaAc and 2.5 volume of ethanol. Leave precipitation mix overnight at -20°C.

**PAUSE POINT **The DNA sample can be left for up to several months at -20°C.

86. Centrifuge the tubes at 14.000 × g for 10 min at 4°C in a micro-centrifuge.

87. Dissolve the pellet in 10-20 μl of water and leave for 20 min at 10°C.

88. Take 1 μl of the fragment (from Step 87) and run 1% gel to ensure that the DNA is not lost.

89. Use 2-4 μg of the digested BAC DNA fragment and 0.01-0.02 μg of the linearized TAR vector for the second round of recombinational cloning (i.e., repeat Steps from 17 to 68).

**CRITICAL STEP **It is worth noting that at this stage, the size of the BAC DNA arrays may be determined directly by CHEF gel electrophoresis without preliminary check on 1% gel (see Figure 1f).

TIMING

Steps 1-7 Preparation of the circular DNA template for rolling circle amplification reaction (RCA): 18 hr

Steps 8-16 DNA amplification reaction by RCA: 13 hr

Step 17 Growth of the culture of the yeast strain: overnight

Steps 18-25 Preparation of competent yeast spheroplasts: 2 hr

Steps 26-30 Transformation of spheroplasts by RCA DNA products along with a TAR cloning vector: 2 hr

Step 30 Colony formation of the His^+ ^transformants: 5 d

Steps 31-32 Incubation of plates with the pooled His^+ ^yeast transformants: overnight

Steps 33-41 DNA isolation from the yeast pools: 4-5 hr

Steps 42-46 Electroporation of yeast DNA into *E. coli *cells: 1.5 hr

Steps 47-62 Miniprep BAC DNA purification from *E. coli *transformants: 3 hr

Steps 63-68 Restriction of BAC DNA, 1% agarose gel and CHEF analyses: 24 hr

Steps 69-89 Further increase of length of the DNA array: two weeks

### TROUBLESHOOTING

Troubleshooting advice can be found in Table 2.

**Table 2:** Troubleshooting table

Steps	Problem	Possible reason	Solution
11	Small size RCA products	DNA cut from the gel is not circular supercoiled	Repeat Step 7; cut the band with the fastest mobility from the gel again
30	Poor transformation efficiency	Spheroplasts are not competent for transformation	Make spheroplasts according to the protocol
30	No yeast transformants	Yeast cells were plated onto wrong medium	Ensure that the medium contains all required nutrients
30	Poor transformation efficiency	TAR vector was phenol/chloroform purified	Vector should be column-purified
30	Poor recombinational cloning	Amount of the TAR vector is more than 40 ng	Check concentration of the vector
30	Poor recombinational cloning	Amount of RCA products is less than 2 μg	Check concentration of the RCA products
30	Poor recombinational cloning	Sequence homology between hooks and the repeats is less than 90%	Change the hooks in a TAR vector
67	No arrays in the vector	Wrong orientation of the hooks in the TAR vector	Orientation of the hooks should correspond to that illustrated in Figure 1c
67	Unstable arrays	Growth of *E. coli *transformants at 37°C	Grow the cells at 30°C
67	Unstable arrays	Growth of *E. coli *culture with good aeration	Grow the cells with a slow shaking
89	No increase in array size after additional recombinational cloning	Cut sites of endonucleases are too far from the ends of the array	Choose endonucleases that cut closer to the end of the array (< 20 bp)

### Anticipated results

The whole procedure, from Steps 1-68, may produce arrays consisting of tandem repeats with a size up to 140 kb. Starting with an amount of template DNA as little as 5-10 ng and with a size of the repeat as small as 340 bp (e.g., alphoid-satellite dimer), it is possible to first amplify the repeat up to 2-5 kb by the RCA reaction (Steps 8-16). For further size increase by recombinational cloning in yeast, each spheroplasts transformation uses 2-4 μg of the RCA products and ~0.01 μg of the linearized TAR vector. Typically, under such conditions, ~200-5000 yeast transformants are obtained (Steps 17-30). Homologous recombination in yeast produces approximately 2% of transformants containing DNA inserts bigger than 15 kb. With less than 1%, the size of the arrays may be as high as 15-30 kb. Thus, an efficient end to end recombination of incoming DNA molecules during yeast transformation results in a recovery of clones with relatively long arrays. After electroporation of DNA isolated from yeast transformants into *E. coli *cells, ~2% of BACs contain DNA inserts bigger than 15 kb and less than 1% of BACs, between 15-30 kb (Steps 42-68). An additional round of recombinational cloning produces approximately 5-10% of clones with a size of arrays 2- to 3-fold larger than the size of the largest array chosen after the first round of recombination (e.g., 20 kb × 3 = 60 kb) (Steps 69-89). Thus, the combination of RCA with a recombinational capture in yeast may increase the original size of a repeat up to 176 times (e.g., 0.34 kb alphoid dimer × 176 = 60 kb) (see Table 1 for more examples).

## Authors' contributions

VL designed the protocol. VNN and NK optimized all steps of the protocol. NL optimized the BAC DNA purification from *E. coli *transformants. VNN, NL, and NK prepared the Figures. NK wrote the protocol. All authors read and approved the final manuscript.

## Competing interests

The authors declare that they have no competing interests.
